# Elastic properties of fly ash-stabilized mixes

**DOI:** 10.1016/j.dib.2015.11.005

**Published:** 2015-11-10

**Authors:** Sanja Dimter, Tatjana Rukavina, Krunoslav Minažek

**Affiliations:** a University of Osijek, Faculty of Civil Engineering, Drinska 16a, 31000 Osijek, Croatia, Europe; b University of Zagreb, Faculty of Civil Engineering, Department of Transportation, Kačićeva 26, 10000 Zagreb, Croatia, Europe

## Abstract

Stabilized mixes are used in the construction of bearing layers in asphalt and concrete pavement structures. Two nondestructive methods: resonant frequency method and ultrasonic pulse velocity method, were used for estimation of elastic properties of fly ash–stabilized mixes. Stabilized mixes were designed containing sand from the river Drava and binder composed of different share of cement and fly ash. The aim of the research was to analyze the relationship between the dynamic modulus of elasticity determined by different nondestructive methods. Data showed that average value of elasticity modulus obtained by the ultrasound velocity method is lower than the values of elasticity modulus obtained by resonant frequency method. For further analysis and enhanced discussion of elastic properties of fly ash stabilized mixes, see Dimter et al. [1].

Specifications Table

**Table:** 

Subject area	*Materials*
More specific subject area	*Dynamic modulus of elasticity E, nondestructive methods*
Type of data	*Table, graph, figure*
How data was acquired	*resonant frequency method (resonance frequency meter 58-E0035) ultrasonic pulse velocity method (ultrasonic pulse velocity tester 58-E0048)*
Data format	*Raw data collected during measurement, calculated, analyzed, tabulated and plotted*
Experimental factors	*The samples of stabilized mixes were prepared and treated as described in Ref.*[1].
Experimental features	*For RF method, vibrations of variable frequency were induced and the resonant frequency that caused the largest deflection was recorded. For UPV method, the time for the ultrasound to pass through the specimen is measured electronically and is registered in the oscilloscope. Both tests were performed on three identically prepared samples. Data were used to calculate the dynamic elasticity modulus E*_*dyn*_.
Data source location	*Faculty of Civil Engineering in Osijek, Croatia*
Data accessibility	*Data with article*

Value of the data

• The data of dynamic modulus of elasticity, derived from both methods in laboratory tests, can be used in analysis and design of the pavement structure in mechanistic-empirical methods, especially for unconventional stabilized materials such as fly ash.
• Comparision between dynamic modulus of elasticity in cement stabilized mixes, obtained from different nondestructive methods, is limited. Data from this research can serve as a benchmark for other researchers in investigation with similar materials.
• This data can be usefull as guidelines to select suitable parameters of stabilized mixes in the development of further experiments (for instance: content of binder, the binder composition, curing time, temperature treatment).

## 1 Data

On three identically prepared samples of stabilized mixes were measured density, resonant frequency, and ultrasonic velocity. For resonant frequency method, vibrations of variable frequency were induced and the resonant frequency that caused the largest deflection was recorded. Longitudinal frequencies of the samples were obtained and used to calculate the dynamic elasticity modulus Edyn. For ultrasonic pulse velocity method, the time for the ultrasound to pass through the specimen is measured electronically and is registered in the oscilloscope. Ultrasonic velocity was calculated and from velocity, dynamic modulus of elasticity was determined. Data of dynamic modulus of elasticity were analyzed, compared, tabulated and plotted.

## 2 Materials and methods

### 2.1 Materials

Stabilized mixes were designed containing sand from the Drava River, and the binder was composed of different proportions of cement and fly ash. The basic granular material, sand, was of uniform size distribution, with a grain size of D50=0.3 mm; it was gray–brown in color, the degree of unevenness was U=d60/d10=2, and the California Bearing Ratio (CBR)=8–12%. Cement CEM II / BM (PS) 32.5N according to EN 197-1:2005 (EN 197-1:2005 Cement—Part 1: Composition, specifications and conformity criteria for common cements) was used as a hydraulic binder. The cement additive used was fly ash. The chemical contents of the fly ash, expressed as percentages of mass of individual components (mass%) are: SiO_2_=53.0, Al_2_O_3_=29.0, Fe_2_O_3_=10.0, CaO=2.5, MgO=1.5, K_2_O=0.2, and Na_2_O=2.0.

Two groups of stabilized mixes were prepared with different quantities of the binder composed of cement and fly ash:

• Group I: mixes with 10% of the binder, of the total sand mass, and
• Group II: mixes with 14% of the binder, of the total sand mass.

The percentage of fly ash in the binder varied in both groups:

• Mixes marked A were 0% fly ash in the binder; (the control mixture)
• Mixes marked B were 25% fly ash and 75% cement in the binder,
• Mixes marked C were 50% fly ash and 50% cement in the binder, and
• Mixes marked D were 75% fly ash and 25% cement in the binder.

Based on the results of previous studies [2], a compaction energy of *E*=1.0 MJ/m^3^ was selected for specimen preparation, at which the maximum dry weight of *γ*_dmax_=1.729 t/m^3^ was achieved at an optimum water content humidity *w*_opt_=12.6%.

Specimens were prepared in cylindrical molds with a 10.0-cm diameter and 20.0-cm height. They were compacted by hand with a Proctor compactor in five equal layers. After preparation, samples were extruded using a hydraulic press from the mold and left for 1 day at room temperature. The samples were afterwards placed in four environmental chambers at curing temperatures of 5°, 15°, 25°, and 35 °C and a constant humidity of 80%. Specimens were cured for 7, 28, 90, and 180 days, after which the tests were performed and the density, resonant frequency, and ultrasonic velocity were measured.

### 2.2 Methods

**Fig. 1:**
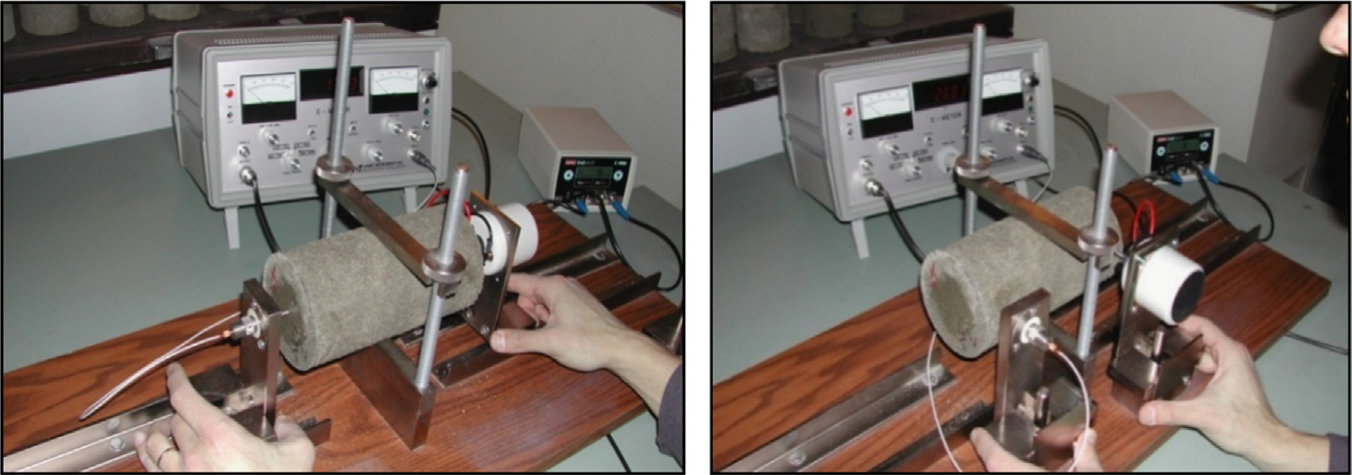
Measuring of longitudinal (left) and torsional (right) resonant frequency.

Determination of the dynamic modulus of elasticity on stabilized mix samples were done using resonance frequency meter 58-E0035 (Fig. 1.) Vibrations of variable frequency were induced and the resonant frequency that caused the largest deflection was recorded.

Longitudinal and torsional frequencies of the samples were obtained and used to calculate the dynamic elasticity modulus *E*_dyn_ (1), dynamic shear modulus *G*_dyn_ (2) and then the Poisson coefficient (3):

(1) $$E_{dyn}=4n^{2}l^{2}p\times 10^{-12},\;\;(MN/m^{2})$$

(2) $$G_{dyn}=4t^{2}l^{2}pF\times 10^{-12},\;\;\left(MN/m^{2}\right)$$

(3) $$\nu =\frac{E}{2G}-1$$

Where *l* is the length of the sample (m), *n* is the longitudinal frequency (Hz), *t* is the torsional frequency (Hz), *p* is the density (kg/m^3^), and *F* is the coefficient that depends on the sample shape (for cylindrical sample *F*=1).

**Fig. 2:**
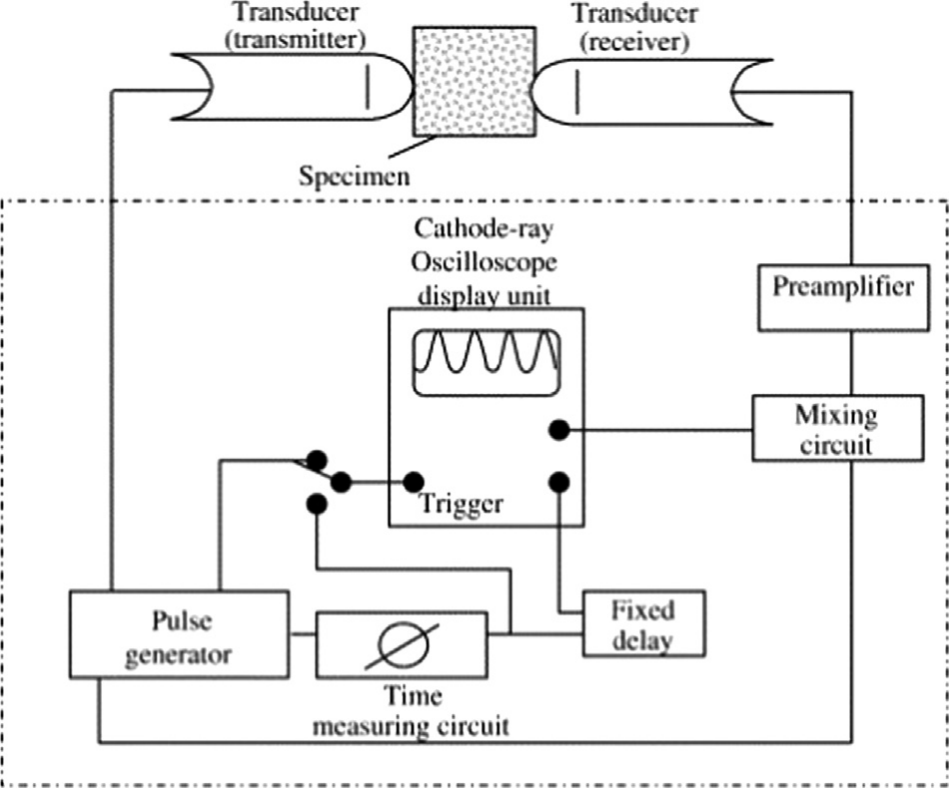
Schematic overview of ultrasonic velocity measurement [3].

Ultrasonic velocity measurement is defined by HRN EN 12504-4 standard and the method is shown in Fig. 2. Determination of pulse velocity on samples were done using ultrasonic pulse velocity tester 58-E0048.

The pulse generator creates electrical impulses of a specific center frequency. The transmitter converts them into elastic waves that propagate through a specimen. The receiver on the other side of the specimen receives the mechanical energy of the propagating waves and turns it into electrical energy of the same frequency. The time (T) for the ultrasound to pass through the specimen is measured electronically and is registered in the oscilloscope. First was calculated ultrasonic velocity (4) and then, from velocity, dynamic modulus of elasticity (5) was determined.

(4) $$v=\frac{L}{T}\;(km/s)$$

(5) $$E_{\mathrm{dyn}}=p\cdot V^{2}\frac{\left(1+\nu )\cdot (1-2\nu \right)}{1-\nu }$$

where *L* is the specimen length (m), *T* is the travel time of ultrasound through the specimen (µs), *V* is the ultrasound velocity (km/s), *p* is the density (kg/m^3^), and *ν* is the Poisson coefficient.

Data of dynamic modulus of elasticity were detail analyzed, compared, tabulated and plotted. Supplementary data associated with this article can be found in the Ref. [1].
